# 

**Published:** 1908-03-15

**Authors:** 


CONTENTS

Event and Comment - - - - - - - - 109

Dentifrices and Mouth-Washes. *By Geo. W. Cook, B.S., D.D.S.* 112

Dental Inspection and Treatment of School Children,
*By R. M. Capon, L.D.S.* 139

Tumors of the Jaws, - - - *By M. Schamberg, D.D.S.* 145

The Cause of Quackery in English Dentistry,
*By R. S. Grant, L.D.S.* 147

Indents - - - - - - - - - - - 150

Announcements - - - - - - - - - 160

